# Bacterial-Viral Interactions in Human Orodigestive and Female Genital Tract Cancers: A Summary of Epidemiologic and Laboratory Evidence

**DOI:** 10.3390/cancers14020425

**Published:** 2022-01-15

**Authors:** Ikuko Kato, Jilei Zhang, Jun Sun

**Affiliations:** 1 Department of Oncology and Pathology, Wayne State University School of Medicine, Detroit, MI 48201, USA; 2 Department of Medicine, Division of Gastroenterology and Hepatology, University of Illinois at Chicago, Chicago, IL 60612, USA; jileiz@uic.edu; 3 Department of Microbiology and Immunology, University of Illinois at Chicago, Chicago, IL 60612, USA; 4 UIC Cancer Center, University of Illinois at Chicago, Chicago, IL 60612, USA; 5 Jesse Brown VA Medical Center, Chicago, IL 60612, USA

**Keywords:** oncoviruses, bacteriophages, *Helicobacter*, sexually transmitted disease, periodontal pathogens, dysbiosis

## Abstract

**Simple Summary:**

Infectious agents, including viruses, bacteria, fungi, and parasites, have been linked to pathogenesis of human cancers, whereas viruses and bacteria account for more than 99% of infection associated cancers. In this review, we discuss how viruses and bacteria interact with each other in a number of biological steps that are linked to the cancer development. We focus on cancers arising from the oral cavity, stomach, intestine, liver, uterine cervix and mammary glands. The interactions may be direct, indirect or synergistic actions in known oncogenic pathways. We found the strength of existing evidence varied substantially from cancer to cancer. We identify gaps in the knowledge for future directions in infection and cancer.

**Abstract:**

Infectious agents, including viruses, bacteria, fungi, and parasites, have been linked to pathogenesis of human cancers, whereas viruses and bacteria account for more than 99% of infection associated cancers. The human microbiome consists of not only bacteria, but also viruses and fungi. The microbiome co-residing in specific anatomic niches may modulate oncologic potentials of infectious agents in carcinogenesis. In this review, we focused on interactions between viruses and bacteria for cancers arising from the orodigestive tract and the female genital tract. We examined the interactions of these two different biological entities in the context of human carcinogenesis in the following three fashions: (1) direct interactions, (2) indirect interactions, and (3) no interaction between the two groups, but both acting on the same host carcinogenic pathways, yielding synergistic or additive effects in human cancers, e.g., head and neck cancer, liver cancer, colon cancer, gastric cancer, and cervical cancer. We discuss the progress in the current literature and summarize the mechanisms of host-viral-bacterial interactions in various human cancers. Our goal was to evaluate existing evidence and identify gaps in the knowledge for future directions in infection and cancer.

## 1. Introduction

Infections are a major preventable cause of cancers [1] and 15.4% of the worldwide cancer cases has been estimated to be attributable to infection [2]. A variety of infectious agents, ranging from viruses and bacteria to parasites, have been linked to pathogenesis of human cancers [2,3]. Among these various taxonomic groups, viruses and bacteria account for more than 99% of infection associated cancers [4], which have been designated to be Group 1 (most established) carcinogenic agents in humans by the International Agency for Research on Cancer (IARC) [5,6]. These include bacteria and viruses. The former includes *Helicobacter (H) pylori* (gastric adenocarcinoma and lymphoma). The latter includes hepatitis virus types B and C (liver cancer), human papillomavirus (HPV) (anogenital and oropharyngeal cancer), Epstein–Barr virus (EBV) (Burkitt lymphoma and nasopharyngeal cancer), human herpes virus type 8/Kaposi’s sarcoma-associated herpes virus (KSHV) (Kaposi’s sarcoma (KS)) and human T-cell leukemia/lymphoma virus type 1(HTLV1) and human immunodeficiency virus-1 (HIV-1, indirectly through immunosuppression) [2,3]. Furthermore, growing evidence suggests that dysbiosis, imbalances in gut resident bacterial populations, can promote carcinogenesis in humans through induction of chronic inflammation, immune-subversion, and production of carcinogenic metabolites, although evidence to support the roles of individual bacterial species is still limited [1,7,8,9].

While extensive data support etiological roles of the infectious agents discussed above, it is well acknowledged that only a fraction of individuals who are infected with or carry these organisms develop malignancies. This suggests the role of co-factors that interact with these infectious agents and potentiate its oncogenic potential. Given that we now know that the human microbiome consists of a wide range of not only bacteria, but also viruses and fungi, there is growing interest in the effects of the microbiome co-residing in specific anatomic niches on modulating oncologic potentials of infectious agents linked to human carcinogenesis. It should be noted, however, that there is a biologically important difference between these two entities, which is that the viruses are intracellular pathogens, while bacteria are generally extracellular pathogens with some exceptions, such as *Salmonella*, *Neisseria* and *Chlamydia*. Furthermore, most known oncoviruses can be integrated in host chromosome to produce their own oncoproteins and directly manipulate host signalling pathways [10].

In this review, we focus on interactions between the two groups of microorganisms, viruses and bacteria, for cancers arising from the orodigestive tract and the female genital tract, in which both bacterial and viral aetiologies have been established or suspected. Consequently, this review does not cover hematologic cancers, i.e., adult T-cell leukemia and lymphoma caused by a human retrovirus HTLV-1 as well as Burkitt’s lymphoma, Hodgkin’s lymphoma and immunosuppression-related non-Hodgkin’s lymphoma caused by EBV [2,6], as knowledge concerning bacterial aetiology or involvement in these cancers is sparse. Likewise, despite several recent studies revealing community structures of bacteriome in benign and malignant kidney tissues and in urine from chronic kidney disease patients [11,12,13], urinary cancer is not discussed here because of little evidence to support viral involvement.

We will examine the interactions of these two different biological entities in the context of human carcinogenesis in the following three fashions: (1) direct interactions, which applies exclusively to bacteriophages and their target bacterial species; (2) indirect interactions, where infection with one group of organisms benefits the other in sustained infection/colonization, reactivation of latent infection or virulence enhancement through modulation of host extra- and intra-cellular environments; and (3) no interaction between the two groups, but both acting on the same host carcinogenic pathways, yielding synergistic or additive effects. Through this review, we aim to evaluate existing evidence and identify gaps in the knowledge to explore for future directions.

## 2. Head and Neck Cancer

### 2.1. Interplay between Periodontal Bacterial Pathogens and EBV, KSHV and HIV

The human oral cavity harbours one of the most diverse microbiomes in the human body; it consists of more than 2000 bacterial taxa and includes viruses, fungi, protozoa, archaea and bacteria, as well as a large number of opportunistic pathogens that are involved in dental, periodontal and systemic diseases [14,15,16]. The oral virome often includes viruses known to be oncogenic to humans, i.e., HPV, EBV, KSHV and HIV-1 [6]. The last three viruses can remain as a latent infection for years, or a lifetime, and are subject to interaction with other oral pathogens.

The oral bacteriome has been associated with the risk of various cancers [17,18,19,20]. While the aetiological role of specific periodontal pathogens in head and neck cancer has not been clearly delineated, oncogenic properties and potential mechanisms of several bacteria that are causally linked to periodontitis have recently emerged. The most extensively studied is *Porphyromonas gingivalis* (Pg) is a member of the red complex, which consist of the three most pathogenic bacteria in periodontitis [21,22]. *Pg* is a non-motile Gram-negative obligate anaerobic intracellular bacterium which expresses an array of virulence factors [16,23,24]. A recent meta-analysis by Xiao et al. presented the summary odd ratio (OR) of 2.16 (95% confidence interval (CI) 1.34–3.47) for the risk of developing various cancers combined, including oral, oesophageal and gastric cancer [25]. Pg may play an important role in carcinogenesis by promoting epithelial mesenchymal transition (EMT), cell proliferation, and tumour invasiveness and by inhibiting apoptosis and adaptive immunity, as summarized in recent reviews [26,27]. Pg has been shown to manipulate several key signalling pathways in carcinogenesis, including overexpression of transcription factors, NF-ĸB, Slug and Snail, ZEB1, vimentin, β-catenin, Matrix metalloproteinases (MMP)-2, 7 and 9, stem cell markers CD44 and CD133 and negative regulators of T and B cell functions, B7-H1 and B7-DC receptors, and downregulation of E-cadherin PI3K/Akt and P53 pathways [26,27].

A few other periodontal pathogens with potential oncogenic properties include *Treponema denticola* (Td), *Aggregatibacter actinomycetemcomitans* (Aa) and *Fusobacterium nucleatum* (Fn). While the association with head and neck cancer in humans has been only demonstrated for Td [25,28], Aa and Fn have positive findings from animal studies in other organ systems [28]. Td and its exotoxin, Dentilisin (chymotrypsin-like protease (CTLP)) have been shown to possess the ability to activate mitogen-activated protein kinase (MAP kinase) signalling pathways controlling cell proliferation and survival, as well as to MMPs promoting immunomodulation and tumour invasion [29,30]. Aa produces cytolethal distending toxins (CDTs) that is functionally and structurally homologous to mammalian DNase I, which can be translocated into the nucleus, and acts as a genotoxin leading to DNA damage [31]. Hematopoietic lineages, particularly lymphocyte, are known to be much more sensitive to CDT than most other cells, inducing rapid apoptosis and thus they also acts as immunotoxins [32,33]. *Fusobacterium* adhesin A (FadA) from Fn can induce an upregulation of the β-catenin pathway and Wnt gene expression, leading to cell proliferation and tumour growth [34]. This is mediated by Annexin A1, acting on the E-cadherin molecular pathway [35].

It has been well documented that patients with acquired immunodeficiency syndrome (AIDS) caused by an HIV-1 infection, have an increased risk of several types of cancer [6]. These include head and neck cancer. A meta-analysis of studies linking HIV-1/AIDS patients to cancer registry data found the risk ratio compared with the general population for oral and pharyngeal cancer of 2.32 (95%CI: 1.65–3.25) [36]. Similarly, the incidence rate ratios of head and neck cancer was elevated in HIV patients in Denmark and in the United States (US), compared with their respective general populations [37,38]. The association is more evident in women who are less confounded by the effect of cigarettes smoking [39,40,41]. HIV-1 is considered to increases the cancer risk indirectly primarily by immunosuppression, allowing for infection with other primary oncoviruses, e.g., EBV, HPV, and KSHV [6], but HIV-1-induced immunosuppression and immune dysregulation also alter oral bacterial compositions and functions. A higher circulating HIV-1 viral load was associated with enrichment by several periodontal pathogens in subgingival plaques, including Pg and Td, which was assessed by the checkerboard DNA–DNA hybridization methods [42]. A 16S rRNA gene sequencing study of salivary microbiome also identified species of *Porphyromonas* that discriminated between pre- and post-antiretroviral treatment samples [43]. These observations support a notion that HIV-1 infection may enhance the presence of potentially oncogenic periodontal bacteria. However, the relationship between periodontal pathogens and HIV-1 has been acknowledged to be bidirectional. It has been documented that HIV-1 infection in CD4-negative mucosal cells is mediated by oral bacteria, e.g., Pg, which trap HIV-1 on the bacterial surface, leading to internalization of HIV-1 virions in host epithelial cells [44]. Moreover, structural components, e.g., lipopolysaccharide (LPS), lipoteichoic acid, and DNA of bacteria, microbial metabolites, such as butyric acid, and soluble mediators produced by gingival resident cells in response to periodontal pathogens, such as granulocyte–macrophage colony-stimulating factor (GM-CSF), have been found with the ability to reactivate latent HIV-1 infection [45,46,47]. Specifically, butyrate, abundantly produced by periodontal pathogens, e.g., Pg and Fn, inhibits histone deacetylases (HDACs), by competing with the HDAC substrate, thus stimulating transcription of many target genes, including proviruses [48,49]. In fact, hypoacetylation of histone proteins by HDAC has been suggested to be involved in the maintenance of HIV-1 latency. Interestingly, a periodontal pathogen, Pg, efficiently reactivates the latent HIV-1 concomitantly with the induction of lysine acetylation of histone H3 and H4 proteins [50]. Synergism between GM-CSF and LPS has been reported in reactivating HIV-1 latent infection, via upregulation of Sp1 transcription factor through the MAP kinase/ERK kinase and p38 kinase pathways, which leads to activation of the HIV-1 LTR in a Tat-independent manner and induction of HIV-1 lytic production [47]. The observed bidirectional associations between HIV-1 and oncogenic periodontal pathogens are likely to facilitate positive feedback that further fuels the development and progression of head and neck cancer.

The involvement of the oral cavity is one of the most common clinical manifestations of KS, especially in HIV-1-positive patients. Dai et al. reported that Pg culture, conditioned by medium or derived lipopolysaccharide, effectively induced KSHV lytic reactivation from infected oral cells and virus entry to host cells during de novo infection through the TLR4 and MAPK p38 pathways [51,52]. Co-incubation of Pg and FN with KSHV-positive lymphoma cells resulted in disruption of viral latency and KSHV reactivation, mediated by activation of the cellular stress-activated mitogen-activated protein kinase (MAPK) p38 pathway, which led to hyperacetylation of histones 3 and 4 KSHV and replication and transcription activator (RTA) expression [53,54]. Increased production of reactive oxygen species (ROS), which is often associated with periodontal pathogen-induced inflammation, has also been shown to mediate KSHV reactivation [55]. Moreover, ROS was able to increase KSHV entry and infection by amplifying the signalling pathways necessary for its efficient entry into virus target cells, dermal microvascular endothelial cells [56]. Furthermore, similarly to HIV-1, reactivation of latent KSHV infection is stimulated by metabolic end products of periodontal pathogen, e.g., butyrate, which is a HDAC inhibitor [57]. Finally, KSHV has been reported to dysregulate macroautophagy [58], which would certainly subvert the clearance of intracellular periodontal pathogen, such as Pg, or translocated oncogenic toxins such as CTLP, CDT and FadA.

Likewise, EBV can be reactivated by metabolic end products of periodontal pathogen, e.g., butyrate, as demonstrated by increased viral lytic mRNAs, BZLF1 and BGLF5, and ZEBRA protein expression in exposed cells, concomitantly increased acetylated histone H3 [57]. In addition, Aa CDT has been reported to reactivate latent EBV infection in EBV positive cells and promote latent infection in non-infected cells via causing genomic instability [59].

It is also noteworthy that several viral oncogenes found in KSHV and EBV manipulate host signalling pathways to acquire one or more hallmarks of cancer [60]. There are overlap in those pathways manipulated by these oncoviruses and the potentially oncogenic Pg, Td, Aa and Fn. For example, tumour-associated inflammation may be induced by both KSHV and Pg through the NFkB pathway, sustained proliferation by KSHV, Pg, Td, and Fn through the Wnt, MAPK and PI3K-AKT pathways, activating tumour invasion by EBV and Pg through EMT, suppression of apoptosis by KSHV and Pg via p53 inactivation, genomic instability by KSHV and Aa through ATM, and immune evasion by KSHV, Td, and Aa through various pathways [60]. Thus, synergistic interactions in such shared pathways between viral and bacterial pathogens in the development and progression of head and neck cancer is plausible. Yet, to date there have been no direct observations from clinical and epidemiological studies to show increased risk of head and neck KS and EB-associated malignancies among patients who carry specific periodontal pathogens or are seropositive to those pathogens.

### 2.2. Interplay between HPV and Periodontal Bacteria

There have been limited evidence to support interaction between HPV and periodontal pathogens in head and neck cancer. Correlation between the presence of HPV and periodontitis remains unclear. A recent qualitative review of 12 studies that tested various biospecimens ranging from gingival tissue/fluid to oral rinse/swab concludes that oral HPV infection may be associated with periodontitis [61]. However, the largest study from the United States involving about 3000 subjects did not confirm this association [62]. Alternatively, Shigeishi et al. reported that the HPV16 E6 viral load in oral rinse samples was associated with an increased total oral bacterial count measured by the dielectrophoretic impedance method [63]. Others found correlation between HPV positivity in oropharyngeal cancer and severity of periodontitis measured by extent of alveolar bone loss [64], suggesting potential interaction between periodontal pathogens or resulting inflammation and HPV. However, other studies [65,66,67] that addressed altered prevalence or composition of oral/periodontal bacteria were too small (i.e., number of HPV positive cases < 10) to draw any reliable conclusion concerning specific bacteria to interact HPV.

### 2.3. Interaction between Bacteriophages and Periodontal Pathogens

Bacteriophages dominate oral viral communities and thus play key roles in shaping oral bacterial ecology [68,69]. Temperate lysogenic phages also can increase bacterial virulence by promoting horizontal gene transfer, and bacteriophage genes have been found to contain a large number of bacterial virulence homologs, suggesting their role as reservoirs for pathogenic gene function [70,71]. In fact, substantial differences in phage compositions, particularly of dental plaques, by periodontal disease status have been documented [68,72]. However, whether these compositional changes in oral bacteriophages lead to overrepresentation of potentially oncogenic periodontal pathogens has not been clarified. Likewise, there are sparse data on bacteriophages that specifically target potentially oncogenic periodontal pathogens. To date, at least one phage that targets each of Td, Aa and Fn have been identified [72]. Among those, only that for Aa has been shown to influence bacterial virulence by increasing release of leukotoxin [73]. In summary, research on oral bacterial-phage interactions is still in its infancy and there have been no directly relevant information concerning their effects on head and neck carcinogenesis.

## 3. Gastric Cancer

### 3.1. Interaction between H. pylori and Bacteriophages

*Helicobacter pylori* is a Gram-negative, microaerophilic, spiral (helical) bacterium colonizing the human stomach, which represents the most common chronic bacterial infection in humans, affecting more than half of the adult population in the world. Importantly, infection by *H. pylori* has been causally linked to gastric adenocarcinoma and gastric B-cell lymphoma [74]. *Helicobacter* *pylori* has a remarkably high level of genetic diversity due to high genetic recombination rates [75,76,77]. *Helicobacter* *pylori* evolves rapidly by both mutation and homologous recombination [75]. This high level of genetic variation not only supports adaptation to the host, but also at the same time enables *H. pylori* to evade host immune responses [78]. Recent advances in the genetic studies of *H. pylori* have shed light on the hypothesis that *H. pylori* and humans co-evolved, which shaped the risk of gastric disease worldwide [79,80]. It has been estimated that modern humans were already infected with *H. pylori* when they left Africa 60,000 years ago [81] and an updated estimate suggests that the history is even older, i.e., 88,000–116,000 years, as old as are anatomically modern humans [82]. New populations of *H. pylori* were derived from ancestral strains originating in Africa and were spread through human migration to and settlement in Asia/Oceania and Europe [81,83,84]. Local adaptation of genomes of *H. pylori* within populations through positive selection has been also reported to potentially account for differential virulence [85].

Phage-like intracellular structures have been detected microscopically in cultures of *H. pylori* shortly after the discovery of this bacterium [86,87]. With advances in sequencing technologies, studies reporting the presence of bacteriophage genomic sequences (prophages) in the genome of *H. pylori* are increasing [88]. To date, more than 70 different prophages have been documented [88,89,90]. These are all DNA bacteriophages and except for a few, detailed taxonomic classification of these phages remains to be elucidated [88,91]. Prevalence of prophages has been reported to be approximately 20% of the isolates of various geographic origins based on the integrase gene [92,93,94], while the prevalence of a specific prophage found in Japanese isolates of *H. pylori*, KHP30, has been reported to very low (1%) [95] and that of PhiHP33 to be much higher, 36% [96]. Because prophages can decay, their absence does not necessarily suggest the bacteria has never been infected by phages. Phylogenetic analysis of strains of *H. pylori* based on two prophage genes and that based on 7 housekeeping genes of *H. pylori* have shown similar geographical segregation [92,93], suggesting co-evolutionary history shared by *H. pylori* and their viruses. It was further speculated that acquisition of prophages probably occurred before speciation of *Helicobacter*, evidenced by the existence of prophage genes in other species of *Helicobacter*, like *H. acinonychis*, *H. felis*, or *H. bizzozeronii* and by a similar genetic synteny of phage genes for distinct lineages [91]. Menard described a novel coding sequence in *H. pylori*, located between JHP1069/HP1141 and JHP1071/HP1143 in the J99 and 26,695 reference strains, respectively, that exhibited high variability in the deduced protein sequences but significant similarity in protein families, e.g., bacteriophage receptor/invasion proteins, indicating a possibility of a strong selection pressure exerted by phages [97]. Disrupted human-pathogen co-evolution, i.e., maladaptation in coevolving species, has been hypothesized to explain disease emergence [98]. Kodaman et al. discovered that among individuals with low African ancestry background that were infected with *H. pylori* of higher African ancestral background had more severe gastric lesions than those infected with *H. pylori* of lower African ancestral background [79]. In vitro coinfection experiments with ancestry (African (AFR) vs. European (EUR))-matched and mismatched *H. pylori* and *Homo sapiens* (HS) gastric cancer cell lines revealed that the host response to *H. pylori* infection was greatly shaped by the human ancestry and that HSAFR showed signs of coevolution with *H. pylori* while HSEUR appeared to be maladapted and preliminarily predicted that mismatched HPAFR × HSEUR will present a higher risk when compared with HPEUR × HSEUR [80]. In summary, coevolution and host adaptation facilitated by bacteriophages may indirectly affect disease risk associated with *H. pylori* infection, depending on host ancestral background.

Furthermore, Vale et al. reported that approximately 40% of the intact *H. pylori* prophages carry insertion sequences (IS) [99]. Subsequent quantitative analysis of recombination events of the prophage soft-core genes showed equally frequent recombination among well conserved housekeeping genes that were usually less prone to recombination. They concluded that phages of *H. pylori* are among the most recombinogenic phages on earth [100], augmenting *H. pylori* high genomic variability itself. Thus, the additional genetic diversity from prophage sequences is likely to provide *H. pylori* with advantages in terms of persistent colonization [88], which is a prerequisite for development of gastric cancer.

While lysogenic phages can mediate horizontal gene transfer between bacteria and contribute to acquisition of virulence factors through transduction and conjugation [88,101,102], limited data are available as to the direct effects of the prophages on the virulence or pathogenicity of *H. pylori*. Kyrillos et al. reported strong statistical correlations between the presence of phiHP33 orthologous genes and two virulence genes for *H. pylori, cagA* and *vacA*, but found no consistent pattern for insertion sites [96]. No functional tests have been conducted on individual phage orthologous genes, but the authors speculated that phage-related genes may carry out the crucial priming for DNA replication in *H. pylori* based on predicted gene annotation [96]. A Japanese group studied behaviours of a KHP30-infected strain of *H. pylori* and novel phage-free derivative strains. Their motility assays showed that the motility of the prophage-free derivatives was considerably decreased compared with that of the parent strain, but the phage-free derivatives showed higher growth rates than the parent KHP30-infected strain [103]. Moreover, they found both parent and derivative strains had alternation in a major virulence gene sequence, which was 22-bp (5′-tcaatgttggaaaaaattccga-3′) insertion at nucleotide position 2471 in the *cagA* gene, resulting in the disruption of the open reading frame of *cagA* due to a frame-shift mutation. As a result, CagA expression was not detected by Western blot in any of these strains, suggesting the prophage infection attenuates the pathogenicity by altering the sequence of *cagA* and the flanking sequences upstream of the *cagA* gene [103]. Certainly, the insertion of temperate phages into the bacterial chromosome can disrupt expression of bacterial genes or their regulatory regions, although phages may be able to restore the disrupted bacterial gene (or regulatory region) by providing a viral copy of that gene [104]. Thus, effects of phage infection on the virulence of *H. pylori* toward gastric cancer risk depend on types of phages infected and their insertion sites into bacterial chromosomes and thus more phages in *H. pylori* need to be characterized for their functions.

In addition, it has been increasingly recognized that phages play an important role in the acquisition, maintenance, and spread of bacterial antibiotic resistance, as shown in *Staphylococcus aureus* [105,106]. Correspondingly, Fan et al. reported that phiHBZC1_1 prophage contains a putative antibiotic resistance gene, HBZC1_17700. The encoded protein showed high similarity to multidrug resistance protein D (emrD) of *Salmonella enterica* [90]. The number of such phage-coded antibiotic resistance genes are expected to grow as more whole genome information for *H. pylori* becomes available.

Finally, while the therapeutic potential of lytic phages have been long recognized, there have been only a few studies reporting lytic phages in *H. pylori*. Two lytic phages (ΦHPE1 and ΦHPE2) were isolated from gastric biopsies, which were classified as the in the *Podoviridae* and *Siphoviridae* families, respectively [107]. The authors tested the lytic activities of these phages on four different *H. pylori* strains, and found three and two of the four strains showed susceptibility to ΦHPE1 and ΦHPE2, respectively [107]. Cuomo et al. described a lytic phage of *Hp* (Hp φ) isolated from gastric biopsies and tested their therapeutic potential alone and combined with lactoferrin (LA) and adsorbed on hydroxyapatite (HA) nanoparticles. They first showed the treatment with the phage alone or combined with LF-HA did not induce cytotoxic effects, while they verified anti-bacterial activity of Hp φ itself and enhanced activity by combination with LF-HA, as these other two compounds stabilized and extended Hp φ activity [108]. Yet, research in this field is still in infancy, far from clinical application.

Overall, the collective evidence suggests that prophages of *H. pylori* have roles in increasing bacterial fitness and persistent colonization and modulating virulence, but there are insufficient data to predict gastric cancer risk for individuals who are infected with *H. pylori* that carries specific types of bacteriophages.

### 3.2. Interaction between H. pylori vs. EBV in Gastric Carcinogenesis

EBV is a γ-herpes virus that causes a life-long latent infection in nearly 90% of adults around the world [6]. The primary infection with EBV occurs earlier in the childhood in low-income countries [6] than in high-income countries due to lower hygiene and sanitary standards, larger family size and overcrowded living conditions [109], which mirrors acquisition of *H. pylori* in high-risk populations. Following primary infection, EBV will enter into the circulating B-cell pool and in certain situations into epithelial cells, which remain in most cases undetected for life in a latent state. EBV-associated malignancies are speculated to result from viral reactivation that is most likely due to interaction with additional cofactors [6], which leads to viral replication and spread to other cells to establish new latent infection where oncogenesis may take place.

Epidemiological data to support causal association between EBV and GC have been limited [6], whereas *H. pylori* is an established cause of non-cardia GC [2], which arises from several stages of premalignant lesions from chronic gastritis, atrophic gastritis to dysplasia [110,111]. However, the EBV genome encodes several oncoproteins required in immortalization and transformation and is an established cause of certain types of lymphomas nasopharyngeal cancer [6]. EBV-positive GC has been thought to be a rare type of GC, the so called lymphoepithelioma [112], but further studies have linked EBV with other more common types of GC [113]. EBV causality in GC is further supported by the fact that in EBV-positive GC, the EBV genome is present in (almost) all cells and is monoclonal and transforming EBV proteins are expressed [6]. EBV-associated GC exhibits a latency pattern intermediate between types I and II with expression of a few oncogenes, such as EBNA-1 and LMP-2A [6,60].

EBV has been detected in 5–18% of gastric carcinomas tissue worldwide [114,115,116], although the positive rates highly depend on the detection methods used. Because EBV positive GC often present distinct histopathological characteristics with more proximal (fundus and corpus) location, different from most gastric adenocarcinomas associated with *H. pylori* with more distal presentation [117], there have been no, or rather, inverse correlations between EPV and positive assays for *H. pylori* at tissue level [118]. Therefore, the majority of GC is not considered to be product of EBV-*H. pylori* interaction, but this does not rule out the possibility of their interplay in gastric carcinogenesis when both agents co-infect the same gastric fields. In fact, a recent in vitro co-culture study suggests that the adhesion of *H. pylori* that possesses pathogenicity island induces the expression of accessory EBV receptors, such as EphA2 and NMHC-IIA, in gastric epithelial cells and thus increase the efficiency of EBV infection [119]. An increasing number of reports suggest bacterial-viral (specifically EBV-*H. pylori*) interactions, where the presence of one of these microorganisms may promote the growth of the other and vice versa and could also increase their virulence [120]. Any concurrent infection may facilitate EBV carcinogenesis through associated immunosuppression and subsequent loss of control of EBV latent infection as well as chronic antigenic stimulation [121].

Because EBV DNA or RNA has been detected in non-atrophic gastritis samples in the absence of *H. pylori* [122,123,124,125], it has been speculated that EBV can induce acute or chronic gastritis. In addition, there are case-reports of gastritis presented with acute EBV-infection [126,127]. In a mouse herpesvirus latent infection model, with C57BL/6J background mice infected with γHV68, which highly resembles EBV infection in humans, infection induced several proinflammatory cytokines, including IL12, TNFα and INFγ [128]. Thus, *H. pylori* and EBV may synergistically exacerbate inflammation. On the other hand, proinflammatory cytokines and other inflammatory mediators produced by *H. pylori*-induced chronic inflammation, such as INFγ and TGFβ, may reactivate latent EBV infection in vivo [6,129]. In vitro, TGFβ1 partially induces reactivation of latent EBV in EBV-infected gastric epithelial cell lines GT38 and GT39 cells, as corroborated by the induction of EBV immediate-early BZLF1 RNA and its protein product ZEBRA and early antigen-D [130]. Monochloramine, an oxidant produced by infection with *H. pylori* in the stomach, can induce the conversion of EBV from the latent to the lytic phase [131]. Serological data in Mexican paediatric and adult populations showed that co-infection of CagA-positive *H. pylori* and EBV had a stronger association with severe mononuclear and polynuclear cell infiltration in gastric mucosa indicating the presence of severe gastritis and increased risk of progression to intestinal-type GC [132,133]. The same group further analysed GC, non-tumour gastric tissue from GC patients and non-atrophic gastritis (NAG) samples, using PCR and in situ hybridization. PCR was positive in 10.67% of GC, 1.3% of non-tumour controls and 8% of gastritis samples. EBV was detected by in situ hybridization in epithelial cells of GC and in a third of NAG samples, suggesting a role for EBV in gastric cancer and early precursor lesions, either as directly oncogenic infecting epithelial cells or indirectly as an inflammatory trigger [122]. In a subsequent study, significant positive trends were found between serum EBV VCA-IgG and IFN-γ, particularly in patients with GC of intestinal type, suggesting the association between EBV and the development of intestinal type of GC and the potential role of IFN-γ in EBV reactivation [134]. Furthermore, persistent activation of Th17 cells appears to be implicated with the gastric inflammation associated with *H. pylori* and EBV co-infection [120]. Th17 cells and their key cytokine, IL-17A, are implicated in the pathogenesis of gastritis induced by *H. pylori* [135], while animal studies have shown that EBV directly induces the secretion of the proinflammatory cytokine IL17 [136].

Another line of evidence indicates that co-infection with EBV may potentiate virulence of the *H. pylori* oncoprotein, CagA [137]. After injected into the host cytosol through type IV secretion system, CagA is activated by host tyrosine phosphorylation by Src family kinases (SFKs) and then by c-Abl and binds to the pro-oncogenic protein tyrosine phosphatase SHP2 [138]. However, Saju et al. found that the SHP2 homologue SHP1 interacts with CagA, and their interaction potentiates the phosphatase activity of SHP1 that dampens the oncogenic action of CagA. In vitro infection of gastric epithelial cells with EBV induced SHP1 promoter hypermethylation, which strengthened phosphorylation-dependent CagA action via epigenetic downregulation of SHP1 expression [137]. In addition, clinical specimens of EBV-positive GC cases also exhibited SHP1 hypermethylation with reduced SHP1 expression [137], suggesting cooperative actions of EBV and *H. pylori* in increasing the oncogenic potential.

Moreover, accumulated epigenetic alterations in gastric mucosa and tumours have been recognized as one of the mechanistic pathways leading to both *H. pylori*- and EBV-induced gastric cancer [139,140,141]. Evidence suggests that the *H. pylori* oncoprotein CagA and EBV oncoprotein LMP2A upregulate major DNA methyltansferases (DNMTs) that control DNA methylation, specifically DNMT1, and DNMT3b [113,142,143]. Accordingly, both EBV and *H. pylori* have been recognized to increase the risk of specific GC molecular subtype denoted as the CpG island methylator phenotype (CIMP), where CpG islands of multiple genes are concurrently methylated [118,144]. Increased promoter methylation in several tumour suppressor genes (TSGs) is found in *H. pylori*- and EBV-associated gastric lesions and cancer with frequencies varied with the infectious agents [140,141,145,146], and thus co-infection is likely to enhance methylating enzymatic activity on respective target genes. Furthermore, using a *H. pylori*-EBV coinfection model with human gastric epithelial cells, Pandey et al. demonstrated that the epigenetic status of EBV-infected cells was modulated by the CagA-positive *H. pylori* infection, leading to upregulation of DNMTs and thus to promoter methylation of CpG-rich islands of cell cycle-, DNA repair, and apoptosis-related TSGs [143]. Hypermethylation of the regulatory regions of these TSGs changed cellular transcription profiles and the microenvironment that favoured oncogenesis and thus coinfection with CagA-positive *H. pylori* stimulated EBV-mediated cell proliferation in this coinfection model system [143].

Another epigenetic component exploited by both EBV and *H. pylori* are micro RNAs (miR). To our knowledge at least three families of miR are targeted in the same direction by both organisms, which include miR200, let-7 and miR155 [141]. Down-regulation of miR200 has been shown to lead to increased proinflammatory cytokine expression by *H. pylori* infection [147] and EMT induction by EBV [141]. Upregulation of miR155 is thought to act as a strong inhibitor of inflammatory response as miR-155 targets IkB kinase-ε (IKK-ε), Sma- and Mad-related protein 2 (SMAD2), and Fas-associated death domain protein (FADD), reducing the release of pro-inflammatory cytokines such as IL-8 and GROα (CXCL1) and attenuating the inflammatory response associated with both *H. pylori* and EBV [141]. Let-7 is considered to function as a tumour suppressor [148] and its downregulation has been observed with both EBV and cells infected with *H. pylori* [141].

Somatic mutations in AT-rich interaction domain 1A (*ARID1A*), which is a subunit of the SWItch/sucrose non-fermentable (SWI/SNF) chromatin remodelling complex and is thought to have a tumour suppression role by regulating chromatin structure and gene expression, have been detected frequently in both EBV- and *H. pylori*-associated GC [113,149,150]. Because homogeneous loss within the tumour is characteristic to EBV-associated GC and because ARID1A mutations are present in chronically inflamed and non-neoplastic gastric mucosa infected by *H. pylori*, abnormality of ARID1A is considered an early event in carcinogenesis [149].

Immune evasion is one of hallmarks of cancer [60] and has been involved in the aetiology of EBV- and *H. pylori*-associated GC. One of the common features of these cancers is increased expression of Programmed cell Death-Ligand 1 (PD-L1; B7-H1 or CD274) [115,151,152], a molecule that can down-regulate immune responses and, consequently and plays an important role in the persistence of chronic infections [151,153]. Expression of PD-L1 is induced by the Th1 cytokines such as IFN-γ and TNF-α [149,153] and in the case of EBV, through a recurrent chromosomal amplification of the 9p24.1 region containing a gene *CD274* that encodes PD-L1 [113]. Hence, coinfection is likely to help each infectious agent to establish persistent colonization as well as cancer development.

A few common host signalling pathways have been described to be dysregulated by both infectious agents. Specifically, activation of PI3K/AKT and ERK/JNK pathways, which leads to increased cell growth and survival and induction EMT as well as increased cell motility, respectively [60,146], has been observed in both EBV- and *H. pylori*-associated GC. In case of EBV, its oncoprotein LMP2A is responsible for these activations, which has been confirmed in both in vitro and in an in vivo transgenic mouse model [146,154,155,156], while CagA is the primary virulence factor of *H. pylori* that activates these pathways [142,146,157,158,159,160].

Finally, despite availability of transgenic mouse models with major virulence genes from *H. pylori* (*cagA*) [146] and EBV (LMP1, LMP2A and EBNA1) [161], the direct effect of dual infection of *H. pylori* and EBV on gastric carcinogenesis has not been reported to date using these models. Yet, as summarized above, potential biological interactions between *H. pylori* and EBV in infected gastric mucosa are supported by manifold mechanisms at genomic, epigenetic, immunological and pathological levels.

## 4. Colorectal Cancer

### Interplay among Gut Microbiome, Bacteriophages and Eukaryotic Viruses

Colorectal cancer (CRC) is the second leading cause of cancer-related deaths in the USA. It progresses in the stepwise process where healthy tissue develops into the precancerous polyps or adenoma in the colon [162]. There are mainly two mechanisms of bacterial-induced tumorigenesis is exerted, including induction of intestinal inflammation and by direct genotoxic activity [163]. Except bacteria, viruses are important components of the microbial community in the colon.

The differences of gut microbiota in CRC patients from healthy individuals, with the enriched phyla Proteobacteria, Fusobacteria, and Lentisphaerae, and reduced phyla *Firmicutes* and *Actinobacteria*, highlights an altered community faecal diversity and abundance [164,165]. Moreover, in CRC patients, the bacterial genera *Fusobacterium*, *Peptostreptococcus*, *Porphyromonas*, *Prevotella*, *Parvimonas*, *Bacteroides*, and *Gemella*, are prominently enriched, while the genera *Roseburia*, *Clostridium*, *Faecalibacterium* and *Bifidobacterium* are decreased [165,166,167]. In a retrospective analysis of patients hospitalized for bacteremia, Kwong et al. found the association of later CRC diagnosis with *Bacteroides fragilis* and *Streptococcus gallolyticus* and other gut microbes that might enter the bloodstream because of intestinal dysbiosis and increased permeability [168]. These associations between bacteremia from specific microbes and subsequent diagnosis of CRC indicate the necessity for a model in which specific members of a microbiota promote colorectal carcinogenesis. Bacteriophages are the main components of the intestinal virome. Bacteriophages shape the bacterial community, promote CRC reducing favourable commensal bacteria, or impair CRC development by reducing the colonization through carcinogenic bacteria [163]. Hannigan et al. evaluated the differences in human CRC virus and bacterial profiles in stool samples. They demonstrated that bacteriophage communities were associated with CRC and potentially impact cancer progression [162]. The diversity of gut bacteriophage community of CRC patients was significantly different from controls, and dysbiosis of gut virome was associated with early- and late-stage CRC by using clinical subgroup analysis [169]. Meanwhile, phages hosting Gram-negative bacteria like enterotoxigenic *Bacteroides fragilis*, *Escherichia coli*, and Fn, have been suggested to have association with CRC development [170,171,172,173]. Moreover, the putative functional role of bacteriophages in the regulation of biofilm production, which has been implicated in colorectal tumorigenesis in the previous studies, further highlighted the critical function of bacteriophages in CRC [174,175]. Additionally, it was reported that bacteriophages could transfer directly into colonic epithelial cells, promote tumour growth and invasiveness in CRC [176,177]. By performing shotgun metagenomic analyses of virome of faecal samples from patients and controls, Nakatsu et al. identified virome signatures associated with CRC by the significantly increased of the diversity of the gut bacteriophage community in patients with CRC compared with controls [169]. As for the association of vitamin deficiency and colorectal cancer [178,179], it is reasonable to believe that VDR-deletion-induced viral and bacterial dysbiosis should have an important role in the CRC. This can be supported by our recent findings that multiple bacteriophages were markedly altered in our conditional VDR knockout mouse model [180]. It is essential to investigate the bacterial alteration and inflammation conditions in the tissue specific VDR deletion mouse in the future.

Bacteria and eukaryotic virus categorically interact in two ways, directly and indirectly interactions. The direct interactions could occur by viral binding to a bacterial cell or viral utilization of a bacterial product, while the indirect interactions include virus damage to underlying epithelial cells, virus displacement of commensal bacteria and virus suppression of the host immune system, all of which often working in concert [181,182]. The majority of direct bacteria-virus interactions are reports associated with viruses infecting the gastrointestinal tract. For instance, in poliovirus infection, the viral receptor binding and viral shedding was increased by the bacterial components, such as lipopolysaccharides (LPS), peptidoglycan, and other *N*-acetylglucosamine-containing polysaccharides [183]. However, the maintenance of phages in an ecosystem (i.e., gut) is different with the eukaryotic virus. It is dependent on its ability to infect a suitable bacterial host who can employ an arsenal of defence mechanisms. When selective pressure occurs in the ecosystem, co-evolution can result in genotypic variants due to mutations at specific sites within both entities, such as the genes encoding phage tail fibres and bacterial surface receptors [184]. Despite the efforts of bacteria to ward off phage attachment, phages can encode enzymes which degrade capsular polysaccharides [184,185,186]. Meanwhile, the bacterial genomes that encode virulence proteins commonly harbour prophages, which could enhance epithelial invasion and exacerbate enteropathy of their bacterial hosts, such as *Salmonella typhimurium* [187]. Using a murine *Salmonella typhimurium* diarrhoea model, researchers have demonstrated that gut inflammation elicits the induction of prophages leading to increased lysogenic conversion and progression of enteric disease [188].

Because of the critical role of intestinal bacteria in the CRC and the unique specificity of phages, a range of therapeutic applications have been developed for the precise delivery of compounds and drugs in some diseases based on the phage-guided nanotechnologies. It has been reported that a phage-guided, biotic-abiotic, hybrid nanomaterial that targeted the pro-tumoral bacteriumFn, was developed for the precise delivery of chemotherapeutic against CRC [187,189]. As we know, the mammalian immune response can be simulated by the presence of whole phage particles and their components, such as genomic DNA/RNA, proteinaceous capsids, and residual bacterial products (i.e., LPS). This was further demonstrated and applied in the CRC study. The researchers in the study used a phage cocktail for the targeted removal of the intestinal pathogen AIEC (adherent invasive *E. coli*), which was associated with CRC. The studied mice with continuous oral administration of the phage cocktail, showed a reduction of intestinal AIEC, downregulation of genes associated with tumour growth and metastasis, and protection from bacteria-exacerbated CRC [187,190]. However, the cell type, phage size, and morphology, which play a major role in phage internalization and impact the efficacy of phage therapy, should be considered when develop a phage therapy [191].

**Figure 1:**
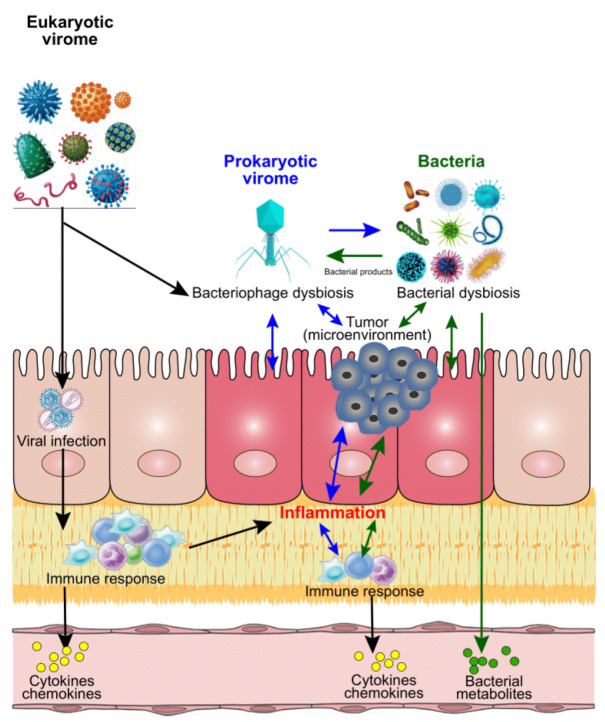
Proposed mechanisms of modulations of bacteria, virus, and CRC. After infection with eukaryotic viruses, the targeted host cells will be used for viral amplification and trigger the host immune responses by secreting cytokines and chemokines. These immune activities could result in inflammation in situ, or in other tissue/organs of the host, which could contribute to the initiation and process of cancer. Similarly, the bacterial infections could contribute to the inflammation via cytokines and chemokines from immune responses, or bacterial metabolites directly. Unlike the eukaryotic viruses, the prokaryotic viruses (mainly bacteriophages) mainly target the bacteria of the host after infection, and further impact on the host bacterial profiles. Meanwhile, the unbalanced bacteria could impact on the viral (both eukaryotic and prokaryotic virus) abundance and diversity through bacterial products. All these invaded organisms could directly or indirectly modulate the host microbial homeostasis, which contributes to the inflammation and tumour microenvironmental dysbiosis and further influence the cancer development and process.

We summarize proposed mechanisms of modulations of CRC development by bacterial-viral interactions in Figure 1. All these findings discussed above suggest that viral (including both eucaryotic virus and bacteriophages) oncogenesis could be a new frontier of CRC microbiome research. Precision medicine depends on the fine-tuning of microbiota functions for which bacteria, virome, and their interactions, may hold the potential for anti-cancer therapies and treatment.

## 5. Liver Cancer

### Interaction between Species of Helicobacter and HCV

Approximately 75% of liver cancer, specifically hepatocellular carcinoma (HCC), has been estimated to be attributable to viral infection, HBV, and HCV [2]. HCV is a single stranded RNA virus belonging to *Flaviviridae* [6], which accounts for about 30% of virus-induced HCC [2]. It remains more challenging in terms of its prevention, due to a lack of a vaccine compared to HBV. HCV, if not cleared, leads to chronic infection causing hepatitis, and then liver cirrhosis and HCC [6]. The HCV genome encodes several structural and non-structural proteins (e.g., core, NS3, NS4b and NS5) that interact with host tumour suppressor and oncogenic pathways, such p53, Rb and Wnt/beta-catenin and MAPK [6], leading to acquisition of all elements of cancer hallmarks [60]. The role of co-factors has been long debated because a small fraction of infected individuals develops HCC. Recent studies suggest that species of *Helicobacter*, including both *H. pylori* and enterohepatic species, may modulate HCV-associated HCC development [192,193,194,195,196,197,198]. Most commonly reported in human series are *H. pylori*, *H. pullorum* and *H. hepaticus. Helicobacter pylori* is a human gastric pathogen that causes gastric cancer and the other two are enterohepatic species with non-human natural hosts (poultry and rodents, respectively) that cause zoonotic infection to humans.

Some of the studies that have addressed co-infection of these organisms, solely relied on serological test for detection of *Helicobacter*. Because of the availability of commercial kits and as it is a known human pathogen, most of these studies focused on *H. pylori*, reporting significantly higher seroprevalence in patients with HCV-associated cirrhosis than in those that were HCV positive but had no cirrhosis [192,193] and increasing seroprevalence with severity of cirrhosis as well as presence of HCC [194,195]. However, these studies were not able to provide any clue as to whether *H. pylori* was present in liver tissue, thereby modulating HCV oncogenesis. As HCV acquisition is not oral, it is unlikely that the presence of *H. pylori* in the stomach affects acquisition of HCV. Thus, the observed associations may merely reflect population level correlation of these two diseases. More importantly, infection with other enterohepatic species of *Helicobacter* cannot be ruled our due to cross-reactivity in serological tests between *H. pylori* and other species of *Helicobacter* [194].

Species of *Helicobacter* have been detected in liver tissues by PCR using genus and species-specific primers in other studies. PCR-positivity to the genus varied with HCV-associated pathologies, from patients negative to HCV (~4%) to HCC (90%) [195,196,197,198]. Within patients with HCC, PCR detection rate was even higher with cancer tissue than surrounding non-tumour tissue [196,199], although one of these studies included both HCV- and HBV-associated HCC [199]. When species were further investigated [196,197,198], the most commonly found was *H. pylori* (at least half or more), followed by *H. pullorum*, which was much less common. Because the liver is not the natural habitat of *H. pylori*, it is possible that its detection in liver represents transient translocation from the stomach. More recent studies focused on another species of enterohepatic *Helicobacter*, *H. hepaticus*, which demonstrated potential of oncogenicity from hepatitis to HCC in animal models [200,201]. The studies in humans to date analysed liver diseases associated with HBV and HCV together. Both Murakami and Yang et al. reported substantially higher seropositivity to *H. hepaticus* in patients with hepatitis virus associated cirrhosis and HCC compared to patients with no viral infection [202,203]. The difference in seropositivity by liver pathology was more apparent with *H. hepaticus* than with *H. pylori* when both were tested simultaneously [203]. One of these studies also employed PCR detection, limited to seropositive samples only to detect *H. hepaticus*. Although the number of tested samples was limited (22 vs. 7), and although the results were not statistically significant, PCR positive rate was twice as high in virus-associated HCC as in patients without viral infection [203]. Despite these positive findings, these PCR-based studies included no more than 40 HCV-associated HCC cases and thus caution should be exercised in generalizing these results.

HCV-transgenic mice have been used to test interaction of HCV with co-infection by species of *Helicobacter*. Fox et al. generated transgenic mice with C57BL/6 background bred with WT C3H. At 18 months, neither HCV transgene expression nor infection with *H. hepaticus* alone was sufficient to increase preneoplastic or neoplastic liver lesions over background levels, but the combination of infection with *H. hepaticus* and HCV transgene expression resulted in a significantly greater incidence and multiplicity of preneoplastic and neoplastic liver foci in males [204]. On the other hand, inoculation of *H. pylori* in the same background HCV transgenic mice did not lead to colonization of the bacterium in the liver and did not promote development of HCC [205]. Using different background mice, Roux-Goglin pointed out that genetic background of transgenic mice affects the synergistic effect between HCV and *H. hepaticus* [206]. Yet, they showed that the multiplicity of lesions (pre and neoplastic) was higher in co-infected mice than those infected with either alone, and multiplicity of the lesions and severity of hepatitis were correlated with presence of the bacteria in the liver [206].

Both gastric and enterohepatic species of *Helicobacter* induce cytotoxicity in hepatocytes *in vitro*, which was particularly potent by *H. hepaticus* [207]. Co-culture of human liver cells with *H. hepaticus* also leads to induction of acute inflammatory responses with increased synthesis of inflammatory mediators and consecutive monocyte activation [208]. In addition, *H. pullorum* increases the expression *of* matrix metalloproteinases (MMPs) in human hepatocytes, which could facilitate metastasis and cell invasion [209]. These toxicity/responses have been mainly ascribed to CDT, a genotoxin as discussed in an earlier section for head and neck cancer, because most enterohepatic species of *Helicobacter*, including *H. pullorum* and *H. hepaticus*, are known to harbour genes encoding CDT, such as CdtB [210]. More details about its chemical and biological properties can be found in a separate review [28]. CDT may not only contribute to genomic mutations also caused by HCV but also promote HCV persistence through suppression of innate and adaptive immunity [211].

By using wild-type strains and CdtB isogenic mutants and by delivering CdtB directly into hepatocyte, Péré-Védrenne et al. demonstrated that CDT from these species of *Helicobacter* induced nuclear factor κB nuclear translocation and proinflammatory responses, T-helper type 17-related and immunomodulatory signatures involved in cancer [210]. Importantly, A/JCr mice infected with an isogenic mutant of *H. hepaticus* lacking CDT activity (CDT mutant) induced chronic hepatitis comparable to wild-type *H. hepaticus* (Hh) infection, however, the CDT mutant-infected mice did not develop hepatic dysplastic nodules, whereas those infected with Hh did [212]. A more recent xenograft mouse models with a hepatic cancer cell line conditionally expressing *H. hepaticus* CdtB revealed a delayed tumour growth and a reduced tumour weight in CdtB-expressing tumours compared to controls, with no difference in proliferating cells undergoing mitosis. However, CdtB intoxication was associated with an overexpression of cytokeratins at the invasive front of the tumour and an increased ploidy, which are hallmarks of endoreplication and aggressiveness in cancer [210]. Thus, these oncogenic properties of CDT may augment oncogenicity of HCV, but details concerning interactions at the molecular level remain to be elucidated. In addition, enterohepatic species of *Helicobacter* have been understudied, which warrants further effort to gain knowledge about its epidemiology and consequences to human health.

## 6. Cervicovaginal Cancer

### 6.1. Interplay between HPV and Other Sexually Transmitted Bacterial Agents

HPV is a small non-enveloped double stranded DNA virus that belongs to the *Papillomaviridae* family. Of this family, the only alpha genus contains mucotropic (verses cutaneotropic) viruses that have been causally linked to anogenital and oropharyngeal cancer in humans [6]. Their oncoproteins, namely E6 and E7, from these high-risk genotypes have been shown to modulate several key signalling pathways, including inactivation of p53, induction of hTERT, binding to PDZ by E6 and inactivation of pRb and related pocket proteins, activation of E2Fs by E7, which leads to inhibition of DNA-damage response, inhibition of apoptosis, inhibition of differentiation, induction of genomic instability, and deregulation of the immune response and cellular energetics and thus fosters immortalization, transformation and tumour progression [6,60]. Although high risk type HPV infections are necessary for development of cervical cancer, again only 0.3–1.2% of women who are infected, develop cervical cancer, while most infection is cleared by the host [60]. This suggests roles of additional factors contributing to persistent HPV infection and/or the disease progression.

Long before the discovery of the link between HPV and cervical cancer, cervical cancer had been thought to be caused by a sexually transmitted agent, due its strong association with sexual history of women and with characteristics of male partners [213,214]. These include *Chlamydia trachomatis* (CT), *Neisseria gonorrhoeae* (NG), *Treponema pallidum*, *Mycoplasma hominis*/*genitalium*, *Cytomegalovirus*, herpes simplex virus type 2 and *Trichomonas vaginalis* [214]. In this article, we discuss first four bacterial pathogens. Co-infection/co-presence of these bacteria with HPV or high-risk HPV has been reported in a number of publications using either DNA-based tests or serology, but their significant statistical interaction has not necessarily been documented [215,216,217,218,219,220,221,222,223]. The association with syphilis/*Treponema pallidum* is generally weak and non-significant [216,217], without evidence of specific bacterial virulence factors that are involved in oncogenesis, and thus likely to reflect a shared risk factor strongly associated with promiscuity.

CT has received research interest as a potential co-factor in HPV-associated cervical carcinogenesis. In a serological study among 118 Portuguese women, seropositivity to CT was significantly higher in women who had high grade squamous intraepithelial lesion (HGSIL) than in control women and, among women with HGSIL, seropositivity was higher in HPV-positive than negative women [215]. PCR-confirmed CT infection was also associated with HPV-positivity in Paraguayan women without any cervical lesions [217]. In a meta-analysis, the summary OR of CT for presence of HPV in cervical smears was reported to be 3.16 (95% CI 2.55–3.90) [224]. Concurrent infection of CT was also a significant predictor of persistent (six months) high-risk HPV infection among female adolescents, Atlanta, Georgia [221]. In an international multi-centric study, CT-seropositivity was associated with a 2-fold increased risk of squamous invasive cervical cancer (OR 2.1; 95% CI, 1.1–4.0) among HPV-positive women [222]. In summary, among various sexually transmitted agents that have been evaluated, CT was most consistently associated with HPV infection. Importantly, gene expression in cervical tissues with or without CT infection showed that CT does modulate the expression of the tumour suppressor genes *caveolin-1* and oncogene *C-myc* in the transformation zone of non-neoplastic cervical tissue [225]. Indeed, *Caveolin-1* expression is down-regulated in cells transformed by the HPV in a p53-dependent manner and restoring *caveolin-1* expression suppresses HPV-mediated cell transformation [226]. The *c-myc* expression in cervical intraepithelial neoplasms (CIN) was closely related to the cell proliferation rate and with progression of CIN [227], whereas HPV E6 oncoprotein is known to interact with and transactivate c-myc in its binding site [228]. Furthermore, cytoplasmic and nuclear NF-B, VEGF-C and survivin were significantly increased in CT-positive/HPV-positive cervical cancer tissues than in CT-negative cervical cancer, suggesting that CT-triggered chronic inflammation could favour the entry and persistence of high risk HPV as well as cell survival and proliferation [229,230].

The association between NG and high-risk HPV infection has been less consistent, but some reports suggest that NG increases moderately the risk of abnormal cervical cytology or HGSIL independently, or in conjunction with, HPV infection [218,219]. NG infection has been shown to induce DNA double strand breaks in human non-tumour vaginal cells transfected with HPV E6/E7. Furthermore, infected cells abolished their expression of the tumour suppressor p53 and induced an increase in the expression of cyclin-dependent kinase inhibitors p21 and p27 [231]. NG also induces a number of proinflammatory cytokines by contact to epithelial cells. Specifically, the binding of NG to epithelial cells leads to the activation of low molecular mass GTPases and sequential protein kinase pathways controlling AP-1 activation. The p21-activated kinases (PAKs) activate the JNK/AP-1 pathway involving unknown MKKK and MKK4 [232]. Morgan et al. also reported, using HPV-positive and negative cell lines. as well as various grades of CIN samples, that HPV E6 mediates the activation of JNK, which drives EGFR signalling to promote proliferation and viral oncoprotein expression [233]. Thus, there is a sound mechanistic basis to support possible interactions of NG with HPV in cervical carcinogenesis.

Genital mycoplasma infections (*Mycoplasma hominis* (MH), *Mycoplasma*, *genitalium* and *Ureaplasma urealyticum* (UU)) have also been studied in conjunction with HPV infection and with cervical cancer. Persistent high risk HPV infection was associated with carriage of *Mycoplasma hominis* with significantly increased OR (8.78, 95% CI1.49–51.6) [223]. A meta-analysis by Liang et al. also reported a significantly increased summary OR of UU for HPV-positive cervical cytology [224]. In proteomic analyses of MH, 29 proteins were predicted to target the nucleus of host cells, including those with the capability to alter normal growth activities [234], 77 target the mitochondria, thereby possibly altering programmed cell death and cellular metabolism [235] and 19 target endoplasmic reticulum of host cells possibly altering protein folding [236], providing underlying biological mechanisms linked to carcinogenesis.

Finally, it is noteworthy that the three sexually transmitted agents discussed above, CT, SG and mycoplasma, are all intracellular pathogens, and thus it is possible that these bacterial pathogens directly interact with HPV oncoproteins, transcriptional machinery or their target host proteins. However, to date, little has been clarified as to the specific bacterial virulence factors or metabolites that account for the altered biological pathways in co-infected cells.

### 6.2. Interaction between HPV and the Vaginal Bacterial Microbiome

The healthy vaginal microbiome exhibits the lowest alpha diversity among all human body habitats, strictly controlled by *Lactobacillus*, which helps maintain low vaginal pH and prevent colonization of potential pathogens with lactic acid production [237,238,239]. Dysbiosis of this community, characterized by reduced populations of species of *Lactobacillus* and increased diversity with anaerobes, on a molecular basis [240] has been linked to the acquisition and persistence of HPV infection, as well as the progression of cervical carcinogenesis [241]. This molecularly defined dysbiosis has also been postulated to be an underlying microbiology of clinical bacterial vaginosis, the most prevalent lower genital tract infection in women of reproductive age throughout the world, which is clinically diagnosed by combinations of three or more of the following four criteria: abnormal vaginal discharge, pH > 4.5, presence of clue cells and fish odour [240,241]. A meta-analysis by Liang et al. showed that clinical diagnosis of bacterial vaginosis is associated with 2.5-fold increased risk of HPV infection and with 1.5-fold increased risk of developing CIN [224]. Tamarelle et al. expanded the meta-analysis incorporating bacterial vaginosis diagnosed microbially by sequencing and reported the summary odds ratio of 1.5 for HPV infection [241]. Depletion of the population of *Lactobacillus* was also confirmed in HPV-infected members of monogynous twins compared with HPV non-infected members of the twins [242].

Lactic acid produced by lactobacilli has been shown to exert the antimicrobial, antiviral and immunomodulatory properties [243]. The ability of *Lactobacillus* to form a multicellular aggregation is one of the proposed mechanisms to explain the protective role of lactobacilli in the human vagina, which mediates the adhesion to epithelial cells and biofilm formation, preventing the entry of pathogens [244]. In addition, certain strains of lactobacilli dampen pro-inflammatory responses elicited by Toll-like receptor (TLR) agonists from cervicovaginal epithelial cells and elicit an increase in anti-inflammatory cytokine, which in part is ascribed to lactic acid [243]. On the other hand, inflammation induced by anaerobes that are increased in bacterial vaginosis [245] impairs mucosa barrier function by affecting tight junction protein expression [246]. This facilitates HPV penetration to the proliferative basal cell layer, which is required to establish chronic infection [247]. While inflammation itself has tumour promoting properties [60,248], increased vaginal polyamine levels, which is linked to tumour progression, have been reported in women with clinically or molecularly diagnosed bacterial vaginosis compared with women without bacterial vaginosis [249,250]. Others have demonstrated that LPS, derived from increased anaerobes in bacterial vaginosis, interfere with tumour suppressors, which are also targeted by HPV oncoproteins, e.g., P53 and E-cadherin, in HPV-positive cervical cancer cell lines [60,251,252,253].

Recently, *Prevotella*, one group of the anaerobes increased in bacterial vaginosis has received research interest for its role in modulating HPV-induced cervical cancer. Normally inhibited by *Lactobacillus*, species of *Prevotella* become abundant when the homeostasis of the vaginal microbial community is disrupted. Species of *Prevotella* may provide nutrients (e.g., ammonia and amino acids), to others, such as *Gardnerella vaginalis* and *Peptostreptococcus anaerobius*, commonly found in bacterial vaginosis. Thus, it has been postulated that *Prevotella* may act as a conductor orchestrating the state of vaginal microbiomes [230,254]. In fact, the genome of *Prevotella* contains loci encoding type VI secretion system, a molecular machinery that transfers effectors from donor to recipient bacteria upon contact and thus acts as important drivers of microbiome composition [255,256]. Most importantly, there has been a clear link between carriages of *Prevotella* and HPV infection in cervicovaginal swabs [242,257]. In addition, species of *Prevotella* express strong enzymic activities in polyamine synthesis, such as arginine decarboxylase, agmatine deiminase, and N-Carbamoylputrescine amidohydrolase [250].

Because these vaginal commensal bacteria are not intracellular pathogens, it is unlikely that they modulate HPV gene expression or interact with its oncoproteins directly within HPV-infected cells. Rather they may facilitate HPV binding to various host molecules for viral internalization [258,259] or counteract antiviral immunity for viral clearance [260]. However, to date little has been known about specific bacterial virulence factors or by-products that modulate host gene expression, metabolism or mucosal innate and adaptive immunity that are involved in viral infection, persistence, or tumour progression. To our knowledge, there have been no animal studies using HPV E6 transgenic mice [261] to test synergistic effects of other sexually transmitted bacterial pathogens.

## 7. Breast Cancer

### Potential Interaction among Retroviruses, EBV and Gut and Milk Bacteriome

Breast cancer affects both males and females, though with 100 times higher incidence in the female population [262,263]. Growing evidence indicated the role of the microbiome, including viruses, in breast cancer [262,264]. There are four viruses that have the most documented oncogenic potential for breast cancer, including mouse mammary tumour virus (MMTV), bovine leukemia virus (BLV), human papilloma viruses (HPVs), and Epstein-Barr virus (EBV, or human herpes virus type 4). The identification of MMTV in human breast cancer has been reported recently [265]. Importantly, the high prevalence of MMTV positive identification in breast cancer indicate a role of MMTV in a subset of breast cancers [264]. Moreover, MMTV infects lymphocytes located in the intestine, and is known to randomly integrate into the human genome located in normal breast epithelial cells [266,267,268]. BLV is a delta retrovirus closely related to the human T cell leukemia virus 1 [264]. Although it is known as the cause of leukemia in beef and dairy cattle, BLV gene were identified by PCR in human breast cancers [269,270,271]. There were 67 (59%) of 114 US breast cancers were positive for BLV when compared with 30 (29%) of 104 normal breast controls (odds ratio of 3) in a case study using the PCR [270]. Interestingly, BLV was identified in 23 (74%) of 31 benign breast biopsy tissues 3–10 years before the development of BLV positive breast cancer in the same patients by collecting the prior benign and later breast cancer specimens from the same individual patients [271]. Negative association between BLV and human breast cancer was also reported [272]. Meanwhile, high-risk HPVs are the cause of cervical cancer and critical for head and neck and other cancers. HPV genes have been identified in breast tumours in over 40 studies conducted in 20 countries with an overall odd of 5.4 [273,274]. High-risk HPVs were found fourfold more prevalent in breast tumours compared to non-cancer controls, although the prevalence of HPV types and associated-risk of cancer varied between countries and regions within the countries [264]. Moreover, EBVs known as the cause of lymphomas have been identified in breast cancer in a wide range of countries using in situ hybridization, immunohistochemistry, in situ PCR, and standard liquid PCR [275]. EBV antibody levels were also reported significantly high in breast cancer patients in a study conducted in south China [276].

As we have discussed before, MMTV is another retrovirus involved in the bacteria-virus interactions and has a critical role in the immune system. Evidence suggested that TLR-4 (Toll-like receptor 4), a pattern recognition sensor that targets LPS, was initiated by the binding of MMTV to bacterial LPS, then activated IL-10 and IL-6, thus allowing the MMTV antigen to evade the immune response and persist in the host [277,278].

Each mother harbours a unique and personalized microbial profile in her breast milk despite the high intra- and inter-individual variability [262,279]. However, the breast milk could also be a potential vehicle for pathogenic virus transmission, include the virus (MMTV, BLV, HPV and EBV) we discussed above. All these viruses or viral DNA have been detected in the breast tissue from breast cancer patients, which may indicate the correlation of these viruses with breast cancer, as mentioned above. More importantly, a study has reported the detection of MMTV in milk from both the reference-group and women who undergo a breast biopsy, but it was significantly higher among women who were at greater than normal risk of breast cancer [280]. Similarly, the infectious BLV has been detected in the dairy cow milk [281]. This was also supported by a 2007 U.S. A Department of Agriculture survey of bulk milk tanks in which they found that 100% of dairy operations with large herds of 500 or more cows tested positive for BLV antibodies [282]. HPV DNA has been detected in the human breast milk by multiple research groups [283,284,285]. Luovanto et al. found that HPV in breast milk is prevalent among the lactation mothers and HPV can also persist in the breast milk which suggested the potential role of breast milk in HPV transmission [286]. Lastly, EBV DNA was also detectable in breast milk involved in several recent studies [283,287,288,289]. More recently, the infectious EBV, not just viral DNA, was found in breast milk supporting the hypothesis that breast milk could be a mean for EBV transmission [290]. Viral activities may impact on the microbiome homeostasis in the local tissue (e.g., breast). However, the potential pathways and mechanisms of vial-microbiome interaction on breast cancer risk need more studies in the future.

## 8. Concluding Remarks

### 8.1. Plausible Potential Mechanisms

**Figure 2:**
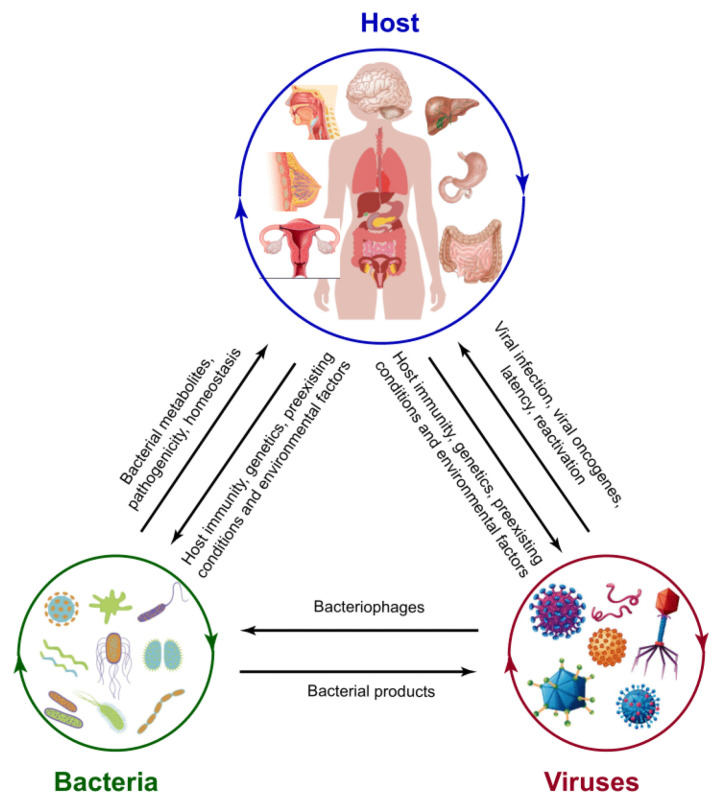
The interrelations of the host, bacteria, and virus. The microbiota of the host, including both bacterial and viral pathogens, could be affected by host immunological activities, genetics, pre-existing conditions, and environmental factors. Bacterial structural components and metabolites and host secretary molecules facilitate viral-bacterial interaction. As one of the viruses, bacteriophages can infect bacteria and have a critical role in shaping bacterial community structure and evolution in virulence. Reversely, bacterial products, such as LPS, could be used by the virus for replication.

We have discussed here a wide range of cancers arising from diverse anatomical sites, which harbour specific microbial communities and are targeted by specific pathogens. Evidence gathered supports several plausible mechanisms that correspond to the types of the interaction proposed at the end of the Introduction. We illustrate the overall view of host-viral-bacterial interactions in Figure 2.

First, viruses, specifically bacteriophages, can shape the evolution of bacterial populations. This may be result in alternation in microbial community structure [291] or virulence of specific pathogens [96]. The former gives rise to dysbiosis that increases the risk of CRC, and examples for the latter include HP in the stomach and *Salmonella enteria* in the intestine.

Second, acquisition or persistence of infection with one group of oncogenic bacteria/viruses can be facilitated by another group infectious agents through multiple mechanisms. Immuno-evasion/suppression in both innate and adaptive immunities induced by both bacterial (e.g., Pg, Aa and Td) and viral pathogens (e.g., HIV) may facilitate acquisition and persistence of infection with another agent. Enteric virus attachment to host cells are enhanced by certain bacterial structural components, such as LPS, peptidoglycan, and other *N*-acetylglucosamine-containing polysaccharides [181,182]. Vaginal bacteria associated with bacterial vaginosis disrupts mucosal barrier, allowing HPV entry to the basal layer where persistent infection can be established.

Third, reactivation of certain viral pathogens (herpes and retro viruses) that stay in latency after initial infection, is induced by bacterial toxins, (CDT), metabolites (e.g., butylate) as well host secretory molecules in response to bacterial infection (GM-CSF, INFγ, TGFβ and ROS).

Fourth, viral and bacterial and pathogens may act synergistically on common pathological and molecular pathways to acquire hallmarks of cancer, reinforcing oncogenic signals from each other. Triggering inflammation is a major pathway exploited by both bacterial and viral pathogens. In fact, chronic inflammation is a prerequisite, premalignant stage for gastric and liver cancers. Beside the primary pathogens (HP and HCV) causing gastritis and hepatitis, proinflammatory actions from additional pathogens are likely to contribute to the progression of premalignant sequences. Furthermore, at a molecular level, viral and bacterial pathogens modulate common oncogenic signalling pathways as well as tumour suppressors within specific anatomic niches. These include Wnt/β-catenin, NFkB, c-myc, PI3K-AKT, JNK, ERK, P53, ATM, and caveolin-1. Some of these effects may be mediated through alterations in host epigenetic properties, including DNA methylation and micro-RNAs, as discussed above. Collaborative actions on the common key signalling pathways by two different entities may be advantageous to surpass threshold, if any, to acquire of specific cancer hallmarks.

### 8.2. Strength of Evidence

We have reviewed a wide range of cancers with potential microbial aetiology to assess existing evidence supporting viral-bacterial interactions in the development cancer, as summarized in Table 1. We found the strength of evidence, as well as types of evidence available, varied substantially from cancer to cancer. Most strong and consistent evidence from epidemiological studies has been published for cervical cancer, though the quality of individual studies was variable. This is not surprising, given a long-known epidemiological association between cervical cancer and STD, but evidence to support roles of individual non-HPV STD agents are not robust, except moderately consistent association with CT. The most likely mechanism is co- or pre-existing bacterial pathogens or overgrowth in establishing persistent HPV infection, which is supported by short-term follow-up studies [221,223]. In this context, clinical intervention to treat STD and bacterial vaginosis in women with premalignant cervical lesions may be warranted.

While it is convincing the bacteriophages have roles in modulating gastric cancer and CRC risk, suggesting a potential for phage-based intervention in modulating cancer risk, the association between known oncogenic viruses and species of *Helicobacter* in the stomach and liver remains to be established due to limited data from studies that assessed pathogen presence at tissue level (not by serology), although there are supporting data from transgenic animal models for liver cancer [204,206]. Despite a multitude of in vitro observations to support underlying mechanisms for the interactions between periodontal pathogens and herpes and retroviruses, there has been sparse information as to whether these periodontal pathogens are more frequently found in vial induced head and neck cancer patients than non-cancer patients or patients with non-virus indued head and neck cancer. Likewise, there is no direct evidence from case-control studies testing tissue level viral gene and microbiome for breast cancer.

### 8.3. Gaps in Knowledge and Future Directions

One of major deficiencies in the current research area is lack of experimental animal models for infection and cancer (except murine MMTV models), due to host specificity of viruses. Infection-associated cancer models are different from chemical carcinogenesis models. Yet, as discussed above, several transgenic animal models have been developed using specific oncogenic viral or bacterial genes and can be a powerful tool to confirm observations in humans in which causal association is often unclear.

The second challenge is the limits in virome research. Despite a growing number of studies reporting virome structures, the data to date are mostly limited to DNA viruses/bacteriophages. It is essential to fully uncover RNA virome community structure in order to dissect complexity of interaction within and between virome and bacteriome. In addition, the vast majority of microbiome research to date has focused on the gut (faecal) microbiome, and thus the role of local tissue resident microbiome in shaping tissue level immune functions warrants further investigation.

The third gap in the current studies is the small sample size. Because tissue procurement procedures are invasive and laborious, earlier studies have often been limited in sample size and there have been no tissue-based prospective studies with cancer endpoints.

To address these issues, large consortium studies as well as cohort studies of high- risk populations, such as patients with AIDS, liver cirrhosis and CIN need to be conceived. Further studies are also necessary to elucidate the effects of anti- proc- and post-biotics and antivirals in prevention of microbially induced cancer. To overcome the limits of experimental animal models, researchers should start to use organoids for modelling infection with pathogens, such as *Salmonella* [292,293,294], viruses [295,296], and gut-microbiota interactions [297,298] to address novel questions in host-microbe interactions, infectious diseases, and the resulting inflammatory conditions. Cancer organoids and human organoids for infection could be further developed for the mechanistic studies and drug screening. A combination of host genetic factors, microbial factors, and environmental and lifestyle factors determine the development of cancers. In addition, the microbiota, including bacteria and viruses, can affect the efficacy and toxicity of chemotherapy, radiotherapy, and immunotherapy. Precise analysis of the change of microbiota and bacterial-virial interactions is essential for prevention, diagnosis and modified treatment in cancer and the eventual clinical outcome.

## Figures and Tables

**Table 1:** Summary of bacterial and viral pathogens that may be jointly involved in the development of human orodigestive and female genital tract cancers.

Target Organ/Cancer	Viruses	Bacteria	Key References *
Head neck (oral)	**Herpes viruses (EBV, KSHS)**	Periodontal pathogens (Pg, Fn, Aa, Td)	[29,57,59]
	HIV-1		[44,46]
Stomach	**EBV**	***H. pylori***	[119,122,137,141,143,149]
	Bacteriophages		[96,97,99,100]
Colorectum	Bacteriophages	**Dysbiotic gut microbiome**	[162,166,169,184]
		**Adherent invasive *Escherichia coli***	[173,190]
		*Salmonella enterica*	[188]
Liver	**HCV**	Enterohepatic and gastric *Helicobacters*	[196,198,200,204,206]
Uterine cervix	**HPV**	*Bacterial STD agents (CT, NG, genital mycoplasma)*	[218,221,224,229]
		*Prevotella*	[242,254,257]
Breast	**Retroviruses (MMTV, BLV)**	Gram-negative bacteria	[262,264,265,270,277,278]

Boldfaced microorganisms: primary oncogenic driver, whose virulence may be augmented by other viral or bacterial coinfection; Non-boldfaced microorganisms: viruses and bacteria that are more likely act as cofactors to the primary oncogenic pathogen or exert indirect effect; Pg: *Porphyromonas gingivalis*, Td: *Treponema denticola*, Aa: *Aggregatibacter actinomycetemcomitans*, Fn: *Fusobacterium nucleatum;* STD: Sexually transmitted disease, CT: Chlamydia trachomatis (CT), NG: Neisseria gonorrhoeae; * Key references do not include all individual citations. For more details, refer to respective sections.
